# Cancer prevalence in a metropolitan HIV clinic

**DOI:** 10.7448/IAS.17.4.19651

**Published:** 2014-11-02

**Authors:** Eleanor Barnes, Cara Saxon, Sameena Ahmad

**Affiliations:** 1 School of Medicine, University of Manchester, Manchester, UK; 2 Department of Sexual Medicine & HIV, University Hospitals of South Manchester, Manchester, UK

## Abstract

**Introduction:**

Morbidity and mortality rates from AIDs defining cancers have fallen significantly since the introduction of highly active antiretroviral therapy (HAART). Patients are now living longer with HIV and are at a greater risk of other HIV- and non-HIV related malignancies. We report what we believe to be the first UK cancer prevalence study in the modern HAART era.

**Methods:**

A retrospective review of electronic clinic letters was performed for all patients currently receiving, and those who had died whilst receiving, their HIV care at our centre. Demographics of patients with pre-cancerous changes, an active or previous cancer were recorded.

**Results:**

There were 438 active patients (369 male, 69 female) and 18 deceased patients (12 male, 6 female) in April 2014. Thirty-six out of four hundred fifty-six (8%) cancer diagnoses were found overall. Thirty-one out of four hundred thirty-eight (7%) diagnoses in active patients and 5/18 (28%) in deceased patients. More than half of those diagnosed with cancer were aged 50 or over (17/31 [55%]). In active patients 17/31 (55%) were AIDs defining cancers, with the most common type of cancer diagnosis overall being Kaposi's sarcoma (12/31 [39%]). There were 5/31 (16%) cases of non-Hodgkin's lymphoma. The most common non-AIDs defining cancer was basal cell carcinoma of which there were 5/31 (16%) cases, followed by squamous cell carcinoma (3/31 [10%]) and testicular cancer (3/31 [10%]). Other cancers included colorectal (2/31 [6%]) and prostate cancer (1/31 [3%]). In all five deceased patients, cancer was the cause of death. There were four acute presentations with an aggressive glioma, Burkitt's lymphoma, an undiagnosed primary lung malignancy and a late diagnosed cervical cancer. The fifth patient died following the recurrence of a transitional cell cancer of the bladder after an initial diagnosis of seven years earlier. Eighteen out of sixty-nine (26%) of females were found to have at least mild dyskariosis on cervical screening. Anal intraepithelial neoplasia was diagnosed in 4/438 (1%) of patients.

**Conclusions:**

Non-AIDS defining malignancies account for almost half of the cancers in our cohort. This number may rise further as patients live longer with HIV. Good communication between oncologists and HIV physicians is paramount to manage the complex interactions of HIV and cancer, increase HIV testing in cancer services and address cancer risk factors in existing HIV patients.

